# Single- versus multi-fraction spine stereotactic radiosurgery (ALL-STAR) for patients with spinal metastases: a randomized phase III trial protocol

**DOI:** 10.1186/s12885-025-13655-6

**Published:** 2025-02-21

**Authors:** Aniket Pratapneni, Daniella Klebaner, Scott Gerard Soltys, Elham Rahimy, Iris Catrice Gibbs, Steven Daniel Chang, Gordon Li, Melanie Hayden Gephart, Anand Veeravagu, Gregory Arthur Szalkowski, Xuejun Gu, Lei Wang, Cynthia Chuang, Lianli Liu, Scott Jackson, Rong Lu, Jillian Adele Skerchak, Kelly Zhe Huang, Samantha Wong, Eleanor Brown, Erqi Liu Pollom

**Affiliations:** 1 Stanford Cancer Institute, Stanford, US; 2 University of California, San Francisco, US; 3 Stanford Medicine, Stanford, US

## Abstract

**Background:**

For patients with spine metastases, stereotactic radiosurgery (SRS) provides excellent local control and pain response. Despite increasing use of this treatment modality, there is no consensus on the optimal dose and fractionation of spine SRS for efficacy and toxicity. We have initiated a single-center phase III randomized trial that compares two dose regimens with similar biological equivalent dose (BED) to determine the isolated effect of SRS fractionation on local control.

**Methods:**

Patients with one to three cervical, thoracic, or lumbar spine metastases spanning no more than two contiguous vertebral levels in need of radiation will be eligible for enrollment. Patients will be assigned 1:1 to receive either 22 Gy in 1 fraction or 28 Gy in 2 fractions. Biased coin randomization will be used to randomly assign patients while balancing the following stratifying variables between the two treatment arms at baseline: gastrointestinal histology (yes/no), paraspinal tissue extension (yes/no), epidural compression (low-/high-grade), and number of sites treated (one to three). The primary endpoint is one-year local control, defined per Spine Response Assessment in Neuro-Oncology (SPINO) criteria. The secondary endpoints include patient-reported health-related quality of life (HRQOL), pain associated with the treated site, vertebral compression fracture (VCF), and two-year local control. Patients will be followed for these outcomes at one to two weeks, one month, three months, and six months after treatment, and every six months thereafter until 24 months after treatment. While on the study, patients will receive routine co-interventions as clinically indicated.

**Discussion:**

The studies published thus far comparing the single- and multi-fraction SRS are lacking long-term local control outcomes and are limited by selection bias as well as single-fraction arms with higher BED, which is correlated with improved local control. Our study will isolate the effect of fractionation by comparing one-year local control in patients treated with single- and multi-fraction SRS with equivalent BED. We anticipate that the results of this, as well as secondary endpoints such as pain response, adverse effects, and quality of life will provide much-needed guidance regarding optimal dose and fractionation for both maximizing local control and minimizing toxicity.

**Clinical trial information:**

NCT#06173401. Approved by Stanford Scientific Review Committee (study ID: BRN0060) on 9/12/2023 and Stanford Institutional Review Board (study ID: IRB-72248) on 11/14/2023

## Background

Spine metastases occur in up to 50% of cancer patients over their lifetime and often result in pain, instability, and neurological compromise [1–3]. Stereotactic radiosurgery (SRS) is a method of delivering high doses of radiation to a target with high precision, over one to a few fractions, and has been shown to have higher rates of pain response and quicker pain relief for bone and spine metastases compared to conventionally fractionated radiotherapy using palliative doses [4–6]. SRS for intact vertebral metastases is also associated with high rates of local control, even for radioresistant histologies [7–9].

Despite the accumulating evidence supporting SRS as an integral treatment for spine metastases and its increasing use in the United States [10], guidance is lacking on the optimal dose and fractionation regimen. Single- and multi-fraction SRS with a range of radiation doses have been used and studied in retrospective and prospective studies. For example, a recent randomized trial from Memorial Sloan Kettering Cancer Center demonstrated lower local failure rates with single fraction SRS (24 Gy in 1 fraction) compared to fractionated SRS (27 Gy in 3 fractions) for metastatic sites, including spine [11]. Two single-institution studies comparing single-fraction SRS (mostly 24 Gy in 1 fraction) and multi-fraction SRS (27–30 Gy in 3 fractions) reported superior one-year local control for single-fraction SRS for renal cell carcinoma and sarcoma spine metastases [12, 13]. However, these discrepancies may be driven by differences in biological effective dose (BED), as the fractionated SRS regimens had lower BED compared to the single fraction regimens. Furthermore, the only level one evidence currently supporting spine SRS is the CC.24 trial which used multi-fraction SRS (24 Gy in 2 fractions) [6]. This trial also showed low rates of vertebral compression fracture (VCF) with this dose regimen (11%), a concern commonly seen with single-fraction SRS [14].

By leveraging radiobiological principles and allowing repair of normal tissue between treatments, fractionated SRS with a sufficiently high BED may be more effective for local control and pose lower toxicity than single-fraction SRS. For example, although single-fraction SRS has historically been used for brain metastases [15], fractionated SRS has allowed the delivery of higher dose to larger metastases or resection cavities resulting in improved local control with lower rates of radiation necrosis [16–18]. A phase III randomized trial comparing single- versus multi-fraction cavity radiosurgery recently completed accrual and we look forward to seeing these results [19].

Additionally, although BED is a useful tool for comparing different radiation regimens, it does not capture all biological factors influencing treatment outcomes. Further, BED calculation is based on the linear-quadratic (LQ) model, which assumes a linear and quadratic relationship between dose and cell killing. This model is well-suited for conventional fractionation but may not accurately reflect the biological effects at very high doses per fraction, as in SRS [20, 21]. Potential benefits of fractionated SRS may include induction of a more favorable immune response, repair of sublethal damage, and ability to more effectively address tumor heterogeneity [22, 23]. These mechanisms may explain studies why studies have shown that fractionated SRS is more effective and associated with less toxicity compared to single-fraction treatments for brain metastases [24] and why fractionated SRS is important to study for spine metastases.

We propose this phase III randomized trial to evaluate the effect of fractionation of SRS on local control of spine metastases by comparing two dose regimens with similar BED (22 Gy in 1 fraction versus 28 Gy in 2 fractions). We hypothesize that multi-fraction SRS, compared to single-fraction SRS, will result in superior local control. Additionally, we will examine the impact of fractionation on pain response, adverse effects including VCF, and HRQOL.

## Methods/design

### Study design

**Fig. 1:**
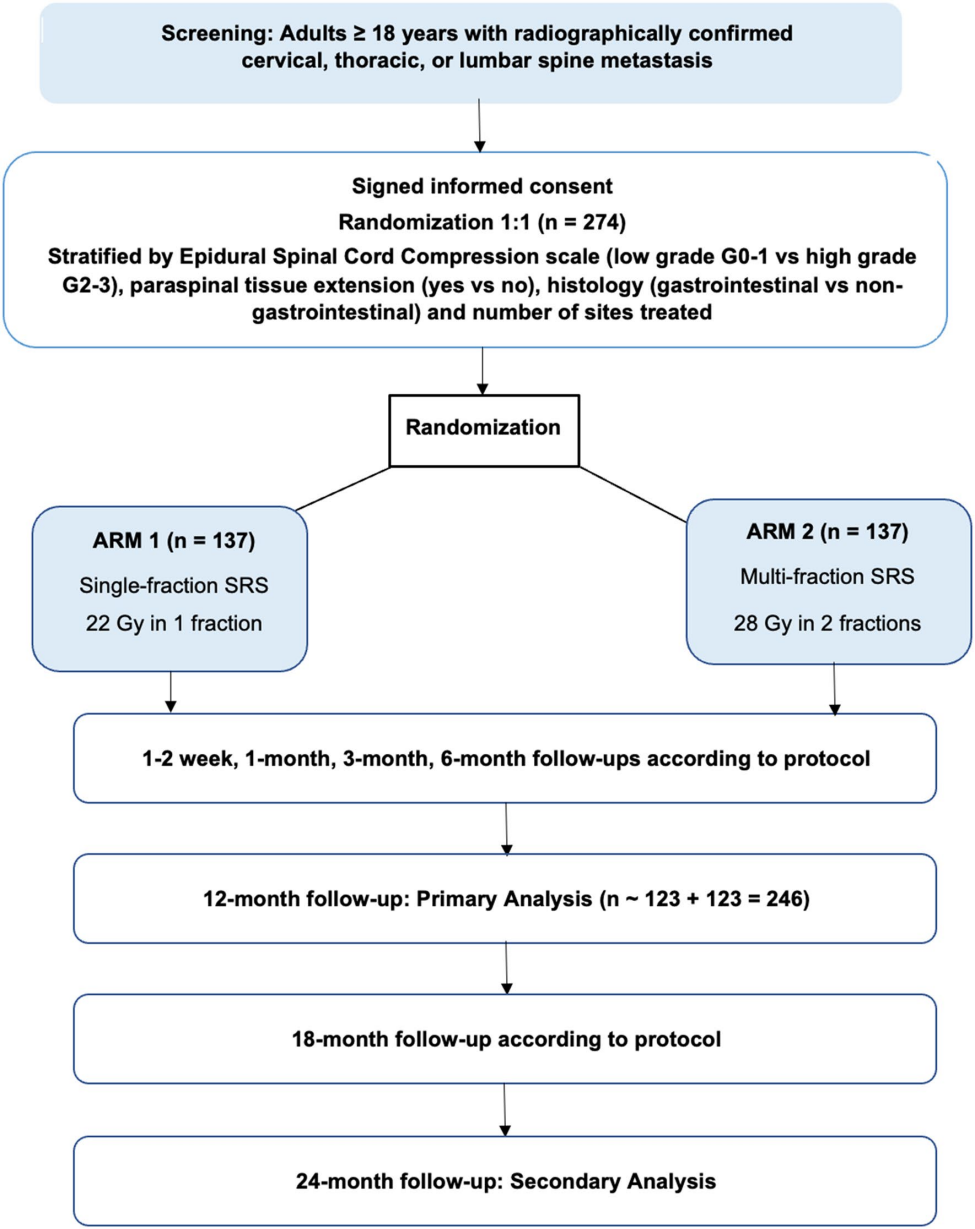
Protocol schema. Phase III trial underway at the stanford cancer institute to compare local control in single-fraction and multi-fraction SRS

This is a prospective, unblinded, randomized, two-arm phase III clinical trial at the Stanford Cancer Institute (Stanford IRB-72248) investigating single- versus multi-fraction spine SRS for patients with spine metastases (NCT#06173401, Fig. 1). Prospective participants will be introduced to the trial at virtual or clinic appointments at the Stanford Cancer Center. Patients may also find information regarding the study online and be referred to Stanford from external sites. Patients will be randomized 1:1 to single- versus multi-fraction SRS. Randomization will incorporate the following strata to minimize imbalances between treatment arms: histology (gastrointestinal versus non-gastrointestinal), paraspinal tissue extension (yes versus no), epidural compression (low- versus high-grade), and number of treated sites (one, two, or three). Biased coin randomization will be done within REDCap to optimize equal allocations to treatment arms within each of the four strata [25].

Upon enrollment, participants will be placed into one of the 24 stratification blocks (for each possible combination of the four stratification variables) and then randomized to a treatment arm using a biased coin design. Based on the current accrual, if there is no size difference between the two arms for that stratification block, the participant will be randomized with a fair coin (50% for each arm). If there is a size difference, the participant will be randomized with a biased coin (66.7% for the arm with fewer participants). Although permuted block designs can be quite effective in eliminating imbalances in stratification variables, they suffer from the disadvantage that at certain time points during the experiment, the experimenter can predict the next subject’s assignment with certainty. By adjusting the probability of assignment to a particular group based on the current imbalance between groups, biased coin designs aim to achieve balance while minimizing the experimenter’s ability to predict the assignment of the next subject.

The evaluation of each stratification variable will be done at the time of enrollment by a treating radiation oncologist or neurosurgeon on the protocol, in the presence of two research coordinators and recorded in the patient’s electronic medical record. Randomization will then be performed within the next 24 h by two research coordinators through the REDCap randomization function (discoverable external module: Extended Randomisation– v9.9.9). During periods when the REDCap system is down for maintenance, free web applications such as the ‘biased-coin randomizer using 4 stratification variables’ can be used as a backup method.

### Study objectives

The primary endpoint of this trial is to determine whether fractionated SRS for spine metastases is associated with improved local control at one year following SRS compared to single-fraction SRS. Secondary endpoints include (1) comparing health-related quality of life following SRS using EQ-5D, EORTC QLQ-C30, and QLQ-BM22 instruments; (2) describing pain associated with treated area at baseline and following SRS; (3) determining one-year grade 2 or higher adverse effects following SRS; (4) determining one-year rate of vertebral compression fracture following SRS; and (5) determining two-year rate of local failure following SRS.

### Ethics, informed consent, and safety

The institutional review board (IRB) of Stanford approved the final protocol (Stanford IRB-72248). All study personnel qualified to conduct the informed consent process will be certified in the protection of human subjects for research. Prior to participation in any study specific procedure, including any invasive screening procedures, all candidates for participation will be provided with a consent form describing the study. This consent form will provide sufficient information to make an informed decision regarding participation. Study participants will provide written informed consent by signing the IRB-approved consent form prior to the conduct of any study-specific procedures. Participants are free to withdraw consent and discontinue participation in the study at any time without prejudice to further treatment.

The protocol, the informed consent, and all forms of information related to the study that will be provided to the subjects (e.g., questionnaires, handouts, written instructions, diaries, advertisements used to recruit participants, etc.) were submitted to, reviewed, and approved by the Stanford Cancer Institute (SCI) Scientific Review Committee (SRC) and the Stanford IRB prior to initiation of the research. Any changes made to the protocol or to the aforementioned participant-facing materials will be submitted as a modification and will be approved by the SRC and IRB of record prior to implementation.

Source documents for all research data will be retained in accordance with all applicable regulations and institutional requirements for data retention. Electronic Case Report Forms (eCRFs) will be developed using the REDCap database system and will be maintained by the clinical research coordinator. Access to eCRFs will be restricted to study staff and appropriately designated individuals and restricted by individual passwords.

The Principal Investigator is responsible for monitoring the conduct of the study including oversight of safety and protocol compliance. On an ongoing basis, the Principal Investigator will review safety data and identify any changes to the research necessary to ensure the appropriate measures and monitoring necessary for participant safety. In addition to the Principal Investigator’s safety monitoring role, the SCI Data Safety Monitoring Committee (DSMC) will conduct data and safety monitoring activities for this study.

### Patient selection and eligibility criteria

Adult patients with one to three radiographically confirmed cervical, thoracic, or lumbar spine metastases in need of radiation treatment will be eligible. Patients are required to have histologically, cytologically, or radiographically confirmed diagnosis of metastatic cancer and ECOG score 0–2. Patients who have had prior surgery or radiation overlapping with the study treatment site will be excluded. Patients with spine metastases of myeloma or lymphoma histologies, spinal instability score (SINS) of 13–18, or lesions causing neurological deficits (strength 1–3 of 5, bladder incontinence, bowel incontinence, and/or bladder retention) will be excluded.

Inclusion Criteria

- Histologically, cytologically, or radiographically confirmed diagnosis of metastatic cancer.
- Age ≥ 18 years.
- Patients who have cervical, thoracic, or lumbar spine metastasis that need radiation treatment.
- Patients will have 1 to 3 separate spinal sites that require radiation treatment. Each spinal site to be treated on trial will span 1–2 contiguous vertebral levels.
- ECOG 0–2 or KPS ≥ 50.
- Negative serum or urine pregnancy test within 2 weeks prior to enrollment for people of childbearing potential or who are not postmenopausal.
- People of childbearing potential and male participants who are sexually active must agree to use a medically effective means of birth control.
- Ability to understand and the willingness to sign (personally or by a legal authorized representative) the written IRB-approved informed consent document.

Exclusion Criteria

- Prior or planned radiation off study within or overlapping with study treatment site.
- Inability to have either an MRI or a CT scan. Patients with pacemaker will be allowed to undergo CT instead of MRI.
- Pediatric patients (age < 18 years old), pregnant patients, and nursing patients will be excluded.
- Histologies of myeloma or lymphoma.
- Patients with strength 1–3 (of 5), bladder incontinence, bowel incontinence, and/or bladder retention that is associated with spinal site to be treated.
- Prior surgery to spinal site intended to be treated with protocol SRS.
- Exclude those sites with SINS 13–18.

### Treatment

All patients will undergo a radiation planning session consisting of CT and stereotactic MRI imaging prior to treatment, followed by delineation of target volumes. The gross tumor volume (GTV) will be defined as gross tumor visualized on CT and MRI in the vertebral body, para-spinal recess, or in the spinal canal. The clinical target volume (CTV) at-risk will be determined based on the location of the GTV based on International Spine Radiosurgery Consortium Consensus Guidelines for Target Volume Definition in Spinal Stereotactic Radiosurgery [26]. An additional margin is recommended in areas of paraspinal extension, and an additional cranio-caudal margin can be considered for areas of epidural extension. The planning target volume (PTV) margin is recommended in the range of 0–3 mm, depending on treatment platform, to account for variations in set-up and reproducibility.

Each eligible PTV will be treated to the prescribed dose according to the patient’s randomization assignment: 22 Gy in 1 fraction or 28 Gy in 2 fractions. The dose is prescribed to the isodose line encompassing at least 95% of the GTV and 90% of the PTV. Ideally, at least 98% of the GTV and 95% of the CTV should be covered by 100% of the prescribed dose if normal tissue constraints can be met (Table 1). Acceptable deviation is 90% coverage of the GTV and 85% of the PTV. The maximum dose (D0.03 cc) to the GTV should be less than or equal to 125%, preferably 120%. The minimum dose (Dmin) to the GTV should be greater than or equal to 15 Gy for single-fraction SRS and greater than or equal to 19 Gy for multi-fraction SRS. Dmin to the GTV can be lower to meet normal tissue constraints.

**Table 1:** Normal tissue constraints for single- and multi-fraction regimens. Multi-fraction dose constraints were calculated to be biologically equivalent to single-fraction dose constraints, assuming alpha/beta of 2

	Single-fraction	Multi-fraction (2 fraction)
Spinal Cord Planning Risk Volume*	V14 Gy < 0.03 cc V10 Gy < 0.35 cc	V19 Gy < 0.03 cc V13.5 Gy < 0.3 cc
Esophagus	V14 Gy < 2.5 cc V12 Gy < 3.8 cc V20 Gy < 0.03 cc	V19 Gy < 2.5 cc V16.5 Gy < 3.8 cc V27.5 Gy < 0.03 cc
Bowel (colon, stomach, small bowel)	V15 Gy < 0.03 cc	V20 Gy < 0.03 cc
Each kidney	Mean < 6 Gy	
Each lung	V20 Gy < 3–5%; V10 < 10%; V5 Gy < 35%; mean < 5 Gy	
Cauda	V16 Gy < 0.03 cc V12 Gy < 0.35 cc	V22 Gy < 0.03 cc V16.5 Gy < 0.35 cc
Nerve root/plexus	Limit D0.03cc < 105–110% of prescription dose	Limit D0.03cc < 105–110% of prescription dose

*Planning risk volume is spinal cord plus 0–2 mm margin depending on treatment platform

Abbreviations: cubic centimeter (cc), Gray (Gy), volume receiving X Gy (VX Gy), maximum dose (D0.03 cc)

Patients will receive routine supportive care as clinically indicated while on this study. Specifically, steroid and pain medication are permitted at the discretion of the treating physician and will be documented (at time of pain assessments when patients report medications). If it is necessary to minimize patient’s anxiety about the treatment and disease condition or for immobilization purposes, medications such as alprazolam or lorazepam are allowed for radiation treatment. Supportive therapy is allowed for medical care of acute radiation symptoms, such as anti-emetics and treatment of mucositis. Data on use of bisphosphonates and similar bone-strengthening agents will be recorded. Chemotherapy is not permitted on the same day as SRS. Immunotherapy, hormone therapy and tyrosine kinase inhibitors are permitted, according to discretion of treating physician.

### Outcome assessments

Patients will undergo MRI and/or CT/PET imaging at 3, 6, 12, 18, and 24 months after treatment to evaluate for local failure of the treated spine site (Table 2). For patients with more than one site treated, local failure will be assessed separately for each spinal level treated. Local control will be interpreted by the radiation oncologist using SPINO criteria [27] as the absence of progression within the treated site on serial imaging. Progression may be defined as: (1) gross unequivocal increase in tumor volume, (2) any new or progressive tumor within the epidural space, or (3) neurological deterioration attributable to pre-existing epidural disease with equivocal or increased epidural disease dimensions on MRI. In cases where radiographic findings are equivocal for true progression versus pseudoprogression or necrosis, repeat imaging and/or biopsy will be performed to differentiate between these outcomes.

**Table 2:** Follow up schedule of outcome assessments. All timepoints are listed from the date of the final fraction of SRS delivered

Outcome Assessment	1–2 Weeks	1 Month	3, 6, 12, 18, 24 Months
AE Evaluation	X	X	X
Numeric Pain Rating Scale	X	X	X
Imaging (PET and/or MR and/or CT)			X
SPINO Assessment for Local Recurrence			X
VCF Assessment			X
HRQOL			X

Abbreviations: adverse event (AE), Spine Response Assessment in Neuro-Oncology (SPINO), vertebral compression fracture (VCF), health-related quality of life (HRQOL)

Grade 2 and higher SRS-associated adverse effects will be collected at baseline and at every follow-up visit until 12 months after treatment. Specifically, VCF will also be assessed with MRI and/or CT imaging. VCF will be classified as any measurable height loss noted on a vertebral body compared with prior imaging [28].

**Fig. 2:**
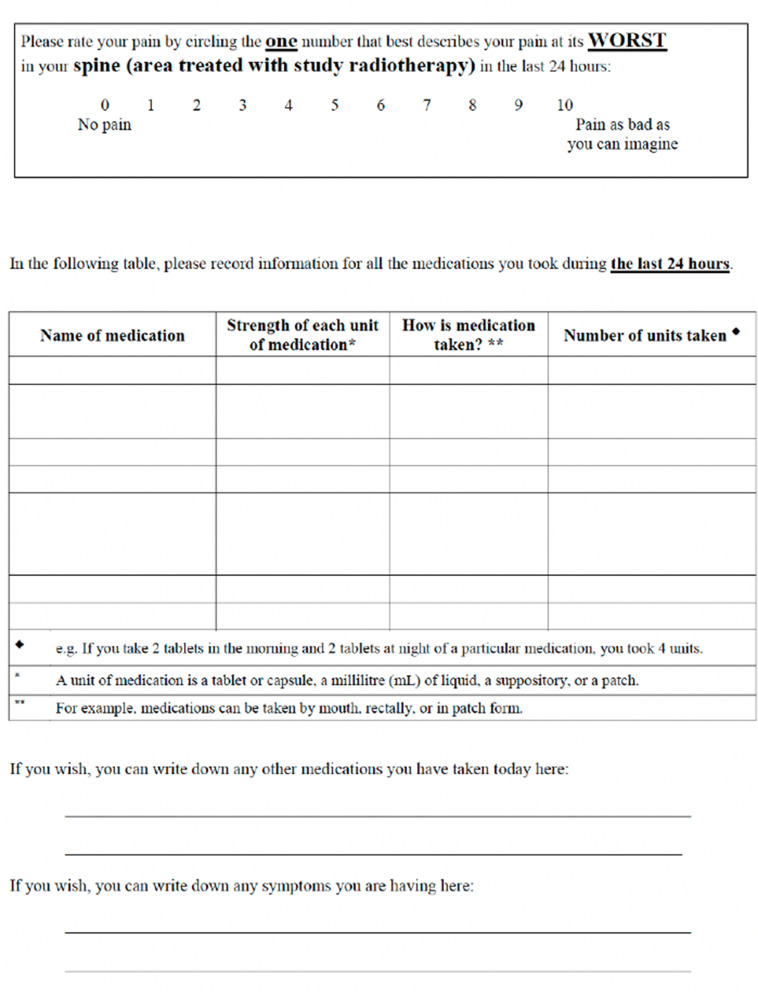
Pain score and medication intake assessment

Pain associated with the treated site will be assessed using a validated numeric pain rating scale [29] at baseline, 1–2 weeks after SRS, and at 1, 3, 6, and 12 months after treatment (Fig. 2). Patients will complete a separate form for each lesion treated with the assistance of a study team member. If two spine sites are within a range of four vertebral bodies, then a single pain form may be used. For three sites within proximity, this pain level categorization will apply unless the clinician and patient can definitively determine the vertebral level associated with pain. Pain response will be reported per the International Consensus Pain Response Endpoint (ICPRE) guidelines [30].

Following treatment, three HRQOL surveys (EORTC QLQ-C30, EORTC QLQ-BM-22, and EQ-5D) will be completed by paper or electronically by the research participants three months after SRS, six months after SRS, and every six months thereafter until 24 months after SRS. Data will be collected and stored in REDCap. As in the CC.24 study, the standard CCTG QoL Response Analysis will be used to categorize patients as having improved, stable, or worsened QOL [31]: the EORTC QLQ-C30’s multi-item scales and single-item measures as well as the EORTC QLQ-BM-22’s four subscales will be linearly transformed to standardize the raw scores to a range between 0 and 100. Then, the minimal clinically important difference for each scale is defined as a change in score of 10 points from baseline. For functional scales, patients will be considered to have improved or worsened if they report a score of 10 or more points greater or lesser (respectively) than baseline, and the reverse classification will be used for symptom scales. Patients whose scores fall within 10 points of baseline will be considered stable.

After completion of treatment, subjects will be followed for two years, or until their removal from study, or death, whichever occurs first.

### Statistical considerations and analyses

#### Sample size determination

Our institutional local failure rate for single-fraction spine SRS is estimated to be 12.5% at one year based on internal data and published retrospective data [32–34]. Retrospective data of 28 Gy in 2 fractions showed one-year local failure rate to be 5.4% [35]. Using a one-sided type one error of 0.10, power of 0.8, and a null proportion of local failures at one year following SRS of 5.4% versus 12.5%, at least 246 patients (123 patients per arm) are required for enrollment to evaluate local control outcomes. Assuming a 10% loss to follow-up rate, a sample size of at least 274 patients is needed to ensure that a minimum of 246 participants will complete one year of follow-up.

#### Timing and impact of interim analyses

The first interim analysis will be performed when approximately 50% of the targeted sample size has reached at least one year of follow-up, estimated as ~ 29 months after the first patient has been randomized and approximately seven months prior to the end of study enrollment. If the lower bound of the 95% confidence interval of odds ratio (local failure in fractionated SRS versus single-fraction SRS) at the time of the first interim analysis is greater than 1 on both patient level and spine site level, then the DSMC may recommend the trial be terminated due to futility.

The second interim analysis (optional, pending the rate of enrollment) will be performed when approximately 70% of the targeted sample size has reached at least one year of follow-up). Based on enrollment assumptions and event rates, it is expected that this will occur approximately 35 months after randomization of the first patient. At this stage, if the efficacy p-value < 0.001 for the odds ratio comparing local control in fractionated SRS versus single-fraction SRS, the recommendation will be made to conclude early superiority of fractionated SRS.

#### Primary and secondary analyses

The population included for primary analysis will consist of all patients who initiated SRS. One-year local failure rates will be reported and compared between the treatment arms. The effect size of fractionation will be reported with a 90% confidence interval. The same approach will later be used to analyze two-year local failure. The time from the end of SRS to the occurrence of local failure as well as the cumulative incidence of local failure will be compared between the treatment arms. Using mortality and VCF as competing risks, the effect of the two fractionation schemes will be evaluated with cause-specific hazard regression. The primary analysis will involve both a per-protocol analysis as well as an intention-to-treat analysis.

For the HRQOL measures, the mean and standard deviations of each of the individual indices at baseline and follow-up time points will be calculated, and scores will be depicted graphically over time. All measures will be compared between the two treatment arms. The effect of the two arms on these outcomes will be evaluated using repeated measure models with a random effect for the subject. Parameter estimates will be provided to represent the effects of both treatment and time. Analysis of variance will be performed to evaluate those effects and their interactions at a significance level of 0.05.

The same statistical approach will be used to evaluate pain response, which will be modeled as a continuous or linear outcome.

To assess VCF and treatment toxicity outcomes, the time from the end of SRS to the first instance of toxicity or VCF will be calculated and compared between the treatment arms, and cumulative incidence will be reported. The effect of the two fractionation schemes will be evaluated with cause-specific hazard regression with mortality and local failure as competing risks. Parameter estimates will be reported with 95% confidence intervals.

For all endpoint analyses, the stratification variables will be adjusted for in multivariate models to eliminate any residual confounding not accounted for by randomization.

#### Reporting and handling of missing data

Missing data will be transparently reported, and its extent and patterns will be analyzed. To handle missing data, if the percentage of missing data is less than 5% across all key variables, the main analysis will primarily use complete cases. If the missing data exceeds 10% at any point during data collection and the observed missing pattern appears to be random, patients with a high degree of missing data will be replaced with newly enrolled patients to control the overall missing data percentages in the final dataset for the primary analysis. Multiple imputation techniques will be employed in sensitivity analyses to validate primary findings. The specific imputation methods will depend on the nature of the data and the mechanism of the missingness, and a detailed account of these processes will be provided in the primary analysis.

#### Assessment of intervention compliance

The adherence of patients to the protocol intervention will be closely monitored and documented. Any instances of noncompliance will be recorded along with the reasons for noncompliance. For the analysis, both intention-to-treat (ITT) and per-protocol analyses will be conducted. The ITT analysis will include all randomized patients as per their assigned group, regardless of the intervention received, to assess the effectiveness of the treatment assignment. The per-protocol analysis will only include patients who fully adhered to the protocol intervention. By comparing these analyses, the impact of compliance on the study outcomes can be assessed.

## Discussion

As cancer patients are living longer with metastatic disease, SRS has become an increasingly important treatment modality to provide durable pain relief and tumor control of spinal metastases. This study will provide guidance for the dose and fractionation of spine metastasis SRS to optimize local control, pain relief, and toxicity outcomes. The novelty of this study lies in its design comparing single- and multi-fraction SRS with equivalent BED. This will isolate the effect of fractionation, permitting a more direct comparison of fractionated versus single-fraction regimens.

Advances in imaging, radiation treatment planning, and radiation treatment delivery systems have significantly improved the ability to deliver precise and high radiation doses in a single or few sessions. Large retrospective series evaluating single-fraction spine SRS have found excellent local control and pain response, compared to non-SRS radiation approaches. A prospective trial at the University of Pittsburgh Medical Center demonstrated a high rate of long-term tumor control (90%) and improvement in pain (86%) among lung cancer patients with painful spinal metastases that were treated with single-fraction radiosurgery (predominantly 20 Gy in 1 fraction) [9]. Another retrospective analysis of 811 spine lesions in 657 patients treated with SRS (predominantly 24 Gy in 1 fraction) at Memorial Sloan Kettering Cancer Center showed that tumor histology (radiosensitive versus radioresistant) did not have a statistically significant effect on the rate of local failure and suggested single fraction SRS can overcome radioresistance to yield low local failure rates (3% crude failure rate) [8].

While fractionation leverages the radiobiological principles of repair, reoxygenation, repopulation, and radiosensitivity to improve the therapeutic ratio of radiotherapy, SRS’s ability to deliver of ablative radiation doses to the tumor while minimizing exposure to surrounding healthy tissues has reduced the need for traditional fractionation. Despite this, there is growing recognition of the potential benefits of fractionation even within the context of SRS to further improve disease outcomes, pain, and treatment toxicity. Existing data comparing fractionated versus single-fraction SRS for spine metastases are limited by selection bias and higher BED used for single-fraction SRS. For example, two single-institution studies comparing single-fraction SRS/SBRT (mostly 24 Gy in 1 fraction) and multi-fraction SRS/SBRT (mostly 27–30 Gy in 3 fractions) found superior one-year local control of spine metastases for single-fraction SBRT in both renal cell carcinoma (95% versus 71%) and sarcoma (91% versus 84%) [12, 13]. However, the single-fraction SRS arms used higher BED and included smaller tumors. A recent randomized trial from Memorial Sloan Kettering Cancer Center suggested lower local failure rates with single fraction SBRT/SRS (24 Gy in 1 fraction) compared to fractionated SBRT/SRS (27 Gy in 3 fractions) for metastases, of which 56% were spine metastases (three-year incidence of local recurrence 6.1 versus 23%) [11]. Although this study did not have the selective biases inherent to the retrospective design of the other studies, the single-fraction SRS arm again used higher BED. Higher BED itself is associated with improved local control: a systematic review and meta-analysis of 37 studies that included 3,237 patients and 4,911 spine metastases treated with single-fraction SRS, multi-fraction SRS and conventional radiation found an approximately 5% increase in local control for each 10 Gy increase in BED [32]. Further, this meta-analysis found that although multi-fraction SRS did not significantly improve one-year local control (82%) compared to conventional radiation (81%), single-fraction SRS did result in a higher rate of one-year local control (93%), suggesting superiority of single-fraction over multi-fraction SRS. However, again the single-fraction SRS treatments included in this study tended to have higher BED: the single-fraction SRS treatments were mainly 20–24 Gy while the multi-fraction SRS treatments were mainly 24–27 Gy in 3 or more fractions. The authors noted that although a schema of 24 Gy in 1 fraction was associated with higher rates of local control than 24–27 Gy in 3 fractions, the 16–18 Gy in 1 fraction regimens with more comparable, but still higher, BED correlated with poorer local control, similar to 24–27 Gy in 3 fractions.

Notably, the only level one data we have so far that supports SRS for spine metastases uses a multi-fraction dose schedule. The CC.24 trial was a multicenter, randomized, controlled, phase II/III study conducted in Canada and Australia that demonstrated superior pain control for spinal metastases treated with two-fraction SRS compared to conventional radiotherapy. This prospective study randomized patients with painful spine metastases 1:1 between SRS (24 Gy in 2 fractions) and conventionally fractionated external beam radiotherapy (20 Gy in 5 fractions) and found a higher rate of complete pain response at three months after treatment with SRS (35% versus 14%) [6]. This contrasts with RTOG 0631, another randomized trial that compared single-fraction SRS (16–18 Gy in 1 fraction) with lower-dose conventional external beam radiotherapy (8 Gy in 1 fraction) for patients with spine metastases. Interestingly, this study found that SRS did not provide superior pain response at three months [36]. However, this trial had baseline imbalances in the treatment arms: the SRS arm contained a higher proportion of the patients with a baseline ECOG performance score of 2, which the authors found as a major predictor of pain response. Furthermore, this study used a lower dose in the SRS arm. This study also did not account for SINS score and thus the contribution of spinal mechanical instability to pain. Another study randomized patients with mostly non-spine bone metastases (where mechanical instability may be less of an issue) 1:1 between similarly low-dose single-fraction SRS (12 Gy for 4 + cm metastases and 16 Gy for smaller lesions) and conventional radiation (30 Gy in 10 fractions) and found that the single-fraction group had more pain responders at two weeks (62% versus 36%), three months (72% versus 49%) and nine months (77% versus 46%) after SRS [37].

Although 24 Gy in 2 fractions is a proven SRS dose, it is still a lower BED than the commonly used single-fraction SRS regimens. However, a recent study compared 28 Gy in 2 fractions with 24 Gy in 2 fractions, and unsurprisingly, found superior local control in the dose-escalated arm: in the 28 Gy cohort, the 6-, 12-, and 24-month cumulative incidences of local failure were 3.5%, 5.4%, and 11.1% respectively, versus 6.0%, 12.5%, and 17.6% in the 24 Gy cohort, respectively (*p* = 0.0075) [35]. The single-fraction equivalent dose to 28 Gy in 2 fractions, assuming an alpha/beta ratio of 10, is ~ 21.5 Gy, which is more comparable to published single-fraction SRS doses, commonly between 16 and 24 Gy [8, 9, 38].

Thus, given that lack of studies using comparable BEDs, which makes it difficult to assess impact of fractionation on outcomes, and given lack of data comparing long-term local control outcomes between different SRS dosing regimens, we propose our trial which will compare 28 Gy in 2 fractions with the biologically equivalent single-fraction SRS dose of 22 Gy. This trial will also compare biologically equivalent single- and multi-fraction dose constraints, assuming an alpha/beta ratio of 2. Using the accepted single-fraction dose constraint for spinal cord of V14 Gy < 0.035 cc and V10 Gy < 0.35 cc [36], the calculated equivalent two-fraction dose constraint for spinal cord is V19 Gy < 0.035 cc and V13.5 Gy < 0.35 cc. This is higher than what was used on CC.24 (Dmax to cord PRV = 17 Gy in 2 fractions) and will additionally provide valuable data on safety of dose-escalation near the spinal cord [6].

We will also evaluate impact of fractionation on toxicities, including VCF. VCF is an important clinical concern following spine SRS, reportedly as high as 39% with single-fraction SRS [14]. VCF is thought to be due to radiation-induced necrosis and fibrosis [39]. Management of VCF may require kyphoplasty or vertebroplasty, but surgical intervention may be indicated if compression causes retropulsion severe enough to elicit neurological symptoms. VCF has been found to be associated with SRS dose in addition to baseline presence of compression fracture > 50% of height, spinal misalignment, and lytic tumor [14, 40–42]. Animal studies suggest that fractionated SRS may be associated with less detrimental effect on microarchitectural, cellular, and biomechanical characteristics in rabbit vertebra than single-fraction SRS [43]. Clinical series seem to support this finding, with VCF observed in only 11% of patients treated with 24 Gy in 2 fractions on the CC.24 trial [6]. The SAFFRON meta-analysis also noted higher VCF rates following single-fraction SRS (19.5%) than multi-fraction SRS (9.6%) [44].

In addition to VCF, our study will also compare important patient-reported outcomes including health-related quality of life and pain response. Pain is a challenging endpoint to assess, especially among patients with metastatic disease, guarded prognosis, and potentially multiple spinal levels to be treated on trial. RTOG 0631’s approach to assessing pain in patients who had multiple spinal levels treated was to record pain at an “index” spine level, defined as the spine lesion with the highest baseline pain score [36]. In contrast, our trial will attempt to assess pain at a more granular level, per lesion rather than per patient. We will collect pain associated with each treated spinal level if patients and providers are able to differentiate location of pain. Like other spine trials, we will assess pain using a validated numeric pain rating scale [24]. To ensure accuracy and completion of pain assessment, research coordinators will assist patients in completing the pain forms in person, over the phone, or via secure videoconferencing.

In addition to pain, there are many validated patient-reported outcome instruments that can be used to capture patient symptom burden and quality of life [45]. We selected instruments used in CC.24 (EORTC QLQ-C30, EORTC QLQ-BM22, and EQ-5D) and RTOG 0631 (EQ-5D) in order to facilitate comparison of these results across trials. Specifically, we included the generic patient-reported outcome instrument, the EQ-5D, so that we can compare our study population to broader patient populations and evaluate cost-effectiveness of different SRS treatments. A benefit of shorter radiation schedules is lower cost as well as increased convenience, particularly for patients who might reside farther away or have transportation barriers. Cost-effectiveness analyses are critical for examining both costs as well as health benefits as measured by quality-adjusted life years (QALY). A cost-effectiveness analysis of CC.24 found that although SRS is associated with higher up-front costs than conventional radiotherapy, the incremental cost-effectiveness ratio (ICER) of the base case was CAD 9,040 per QALY gained, indicating that in the long-term, SRS was more likely to be cost-effective than conventional radiotherapy arm [46].

In conclusion, studies like this trial are needed to reconcile the heterogeneity in existing SRS approaches and provide guidance on the optimal balance of radiation dose and number of fractions to maximize local control while minimizing toxicity. Data on patient-centric endpoints such as pain response, adverse effects, and quality of life, in addition to local control, are crucial for comprehensively evaluating SRS outcomes. We anticipate the results of this trial will inform healthcare decision-making that is evidence-based, patient-centered, and cost-effective.

## Data Availability

No datasets were generated or analysed during the current study.

## Abbreviations

BED: Biological effective dose
CAD: Canadian dollars
CCTG: Canada Clinical Trials Group
CTV: Clinical tumor volume
D0.03cc: Maximum dose
Dmin: Minimum dose
DSMC: Data Safety Monitoring Committee
ECOG: Eastern Cooperative Oncology Group
eCRF: Electronic case report file
GTV: Gross tumor volume
Gy: Gray
HRQOL: Health-related quality of life
ICER: Incremental cost-effectiveness ratio
ICPRE: International Consensus Pain Response Endpoint
IRB: Institutional Review Board
OAR: Organ at risk
PTV: Planning tumor voume
QALY: Quality-adjusted life year
RTOG: Radiation Therapy Oncology Group
SBRT: Stereotactic body radiation therapy
SCI: Stanford Cancer Institute
SINS: Spinal Instability Neoplastic Score
SPINO: Spine Response Assessment in Neuro-Oncology
SRC: Scientific Review Committee
SRS: Stereotactic radiosurgery
USD: US dollars
VCF: Vertebral compression fracture
